# Variance components of sex determination in the copepod *Tigriopus californicus* estimated from a pedigree analysis

**DOI:** 10.1002/ece3.9997

**Published:** 2023-05-02

**Authors:** Jean M. L. Richardson, Heather J. Alexander, Bradley R. Anholt

**Affiliations:** ^1^ Bamfield Marine Sciences Centre Bamfield British Columbia Canada V0R 1B0; ^2^ Department of Biology University of Victoria Victoria British Columbia Canada

**Keywords:** animal model, polygenic sex determination, sex ratio, threshold trait

## Abstract

Extensive theory exists regarding population sex ratio evolution that predicts equal sex ratio (when parental investment is equal). In most animals, sex chromosomes determine the sex of offspring, and this fixed genotype for sex has made theory difficult to test since genotypic variance for the trait (sex) is lacking. It has long been argued that the genotype has become fixed in most animals due to the strong selection for equal sex ratios. The marine copepod *Tigriopus californicus* has no sex chromosomes, multiple genes affecting female brood sex ratio, and a brood sex ratio that responds to selection. The species thus provides an opportune system in which to test established sex ratio theory. In this paper, we further our exploration of polygenic sex determination in *T. californicus* using an incomplete diallel crossing design for analysis of the variance components of sex determination in the species. Our data confirm the presence of extra‐binomial variance for sex, further confirming that sex is not determined through simple Mendelian trait inheritance. In addition, our crosses and backcrosses of isofemale lines selected for biased brood sex ratios show intermediate phenotypic means, as expected if sex is a threshold trait determined by an underlying “liability” trait controlled by many genes of small effects. Furthermore, crosses between families from the same selection line had similar increases in phenotypic variance as crosses between families from different selection lines, suggesting families from artificial selection lines responded to selection pressure through different underlying genetic bases. Finally, we estimate heritability of an individual to be male or female on the observed binary scale as 0.09 (95% CI: 0.034–0.14). This work furthers our accumulating evidence for polygenic sex determination in *T. californicus* laying the foundation for this as a model species in future studies of sex ratio evolution theory*.*

## Graphical abstract

We use an incomplete diallel crossing design and the “animal model” for analysis of the variance components of sex determination in *Tigriopus californicus*. We confirm the presence of extra‐binomial variance for sex, demonstrate that crosses and backcrosses of isofemale lines selected for biased brood sex ratios show intermediate phenotypic means, as expected if sex is a threshold trait determined by an underlying “liability” trait controlled by many genes of small effects, and estimate heritability of an individual to be male or female on the observed binary scale as 0.09 (95% CI: 0.034–0.14). This work furthers our accumulating evidence for polygenic sex determination in *T. californicus*.

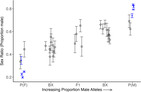

## 1 INTRODUCTION

Sex determination and sex ratio are intrinsically linked. In organisms where sex is determined solely by sex chromosome, the sex ratio for the offspring of a parent is determined on the basis of Mendelian inheritance and can be predicted simply from a binomial distribution (Krackow et al., 2002). Where multiple sex factor genes or other genes that affect sex tendency are present, sex ratio of offspring may be less straightforward, as has now been observed in a variety of fish species (Faggion et al., 2019; Liew et al., 2012; Vandeputte et al., 2007). In addition, environmental effects, including maternal effects, can modify sex tendency in an individual (Bull, 1985; Bull et al., 1982; Radder et al., 2008; Sarre et al., 2004).

Historically, sex determination tended to be labeled as genetic (GSD) or environmental (ESD), but it is increasingly clear that this traditional view of sex determination is unreasonably simple (Beukeboom & Perrin, 2014; Uller & Helanterä, 2011). A variety of recent studies suggest that in species with sex chromosomes, sex of individuals can be strongly influenced by modifier autosomal genes and/or environmental effects. Notably, this has been observed in a variety of fish when reared in captivity, with aquaculturists often intentionally modifying population sex ratios to increase production of the more profitable sex (Vandeputte & Piferrer, 2019; Zhou et al., 2020). In sea bass, Vandeputte et al. (2007) showed that both a polygenic model with environmental variance and a two‐locus gene model with environmental variance fit data from a crossing design.

Regardless of the sex‐determining mechanism, theory predicts that, where male and female offspring are equally costly to produce, population sex ratio should be stable at 50% males and females (Fisher, 1930; Karlin & Lessard, 1983; Shaw, 1958; Shaw & Mohler, 1953) and unstable at biased sex ratios. Further, theory predicts polygenic determination of sex should never be stable. Yet the harpacticoid copepod, *Tigriopus californicus*, a benthic species inhabiting high‐intertidal pools along the west coast of North America, is well documented to have highly variable sex ratios (Ar‐Rushdi, 1958, 1963; Voordouw et al., 2005; Voordouw & Anholt, 2002b) that, while affected by environment, are also highly heritable in a manner that indicates polygenic inheritance (Alexander et al., 2014, 2015).

The developmental process of sex determination in *Tigriopus californicus* is not known, but males engaging in precopulatory mate‐guarding will clasp juveniles as early as copepodite stage CII (Burton, 1985). While males clasped both male and female juveniles in a behavioral lab study, male‐juvenile pairs of *T. californicus* collected both in the field and from lab cultures indicate high accuracy of pairing with females; all 44 juveniles from sampled pairs that survived to adulthood for sex determination were female in a study by Tsuboko‐Ishii and Burton (2017). Thus, sex is presumably determined by this stage. (Sexes cannot be differentiated morphologically until copepodite stage CIV.) Work on delineating sex determination and sex ratio processes in *T. californicus* is ongoing. Foley et al. (2013) crossed isofemale lines from two different populations and found that sex and mitochondrial background are significantly associated with genetic markers in nine of 12 chromosomes. In previous studies (Alexander et al., 2014, 2015), we selected on brood sex ratio to create male‐ and female‐biased lines, showing that brood sex ratio responded to artificial truncation selection (Alexander et al., 2014). Using a California population of *T. californicus* with a published linkage map of SNP markers, we conducted crosses between that population (Foley et al., 2011) and a British Columbia population to identify quantitative trait loci (QTL) associated with brood sex ratio, finding that at least six QTL for brood sex ratio exist on five different chromosomes (Alexander et al., 2015).

Domestic animal breeders have long used pedigrees and estimated breeding values to select for desirable traits in animals. This best linear unbiased prediction (BLUP) approach uses a mixed model with pedigree information used to estimate the additive genetic covariance among individuals and is commonly known as the “animal model” (Lynch & Walsh, 1998). In its simplest form, it estimates the phenotypic value of an individual as the population mean value plus the additive genetic value of the individual plus residual variance. The mixed model approach combined with Bayesian analytic techniques provides a robust and useful tool for estimating genetic components of complex traits.

Here, we take advantage of the selected lines from the local population to conduct a diallel cross followed by backcrosses to selected lines to further explore polygenic sex determination in *T. californicus*. We measure extra‐binomial variation for sex and compare observed and expected mean brood sex ratio values in crosses among selected lines. Finally, using pedigree data for F1 crosses and F2 backcrosses, we estimate heritability of sex in an individual.

## 2 METHODS

Copepods were collected from high intertidal pools at Aguilar Point, British Columbia (48°51′28″ N, 125°09′38″ W). Samples from five pools were collected in September 2009 and transported directly to the laboratory at nearby Bamfield Marine Sciences Centre. From this initial group, two selection lines were established using truncation selection: male‐biased and female‐biased. For the male‐biased line only females that produced the most male‐biased brood sex ratios (BSRs) were allowed to breed in the next generation and for female‐biased lines only females that produced the most female‐biased BSRs were allowed to breed in the next generation (see Alexander et al., 2014 for complete details). At the end of six generations this resulted in a male‐biased line with a mean (±SD) proportion male BSR of 0.64 ± 0.029 and female‐biased line with a mean (±SD) proportion male BSR of 0.35 ± 0.031 (Alexander et al., 2014). While in the lab, *Tigriopus* were housed in filtered sea water (sea water drawn from 15 m deep in the ocean and filtered through a 1‐micron filter), held at room temperature (20°C) and fed ad libitum a mixture of ground TetraMin® and Spirulina flakes (50:50 by weight) suspended in filtered sea water at a ratio of 0.01 g/mL.

**FIGURE 1:**
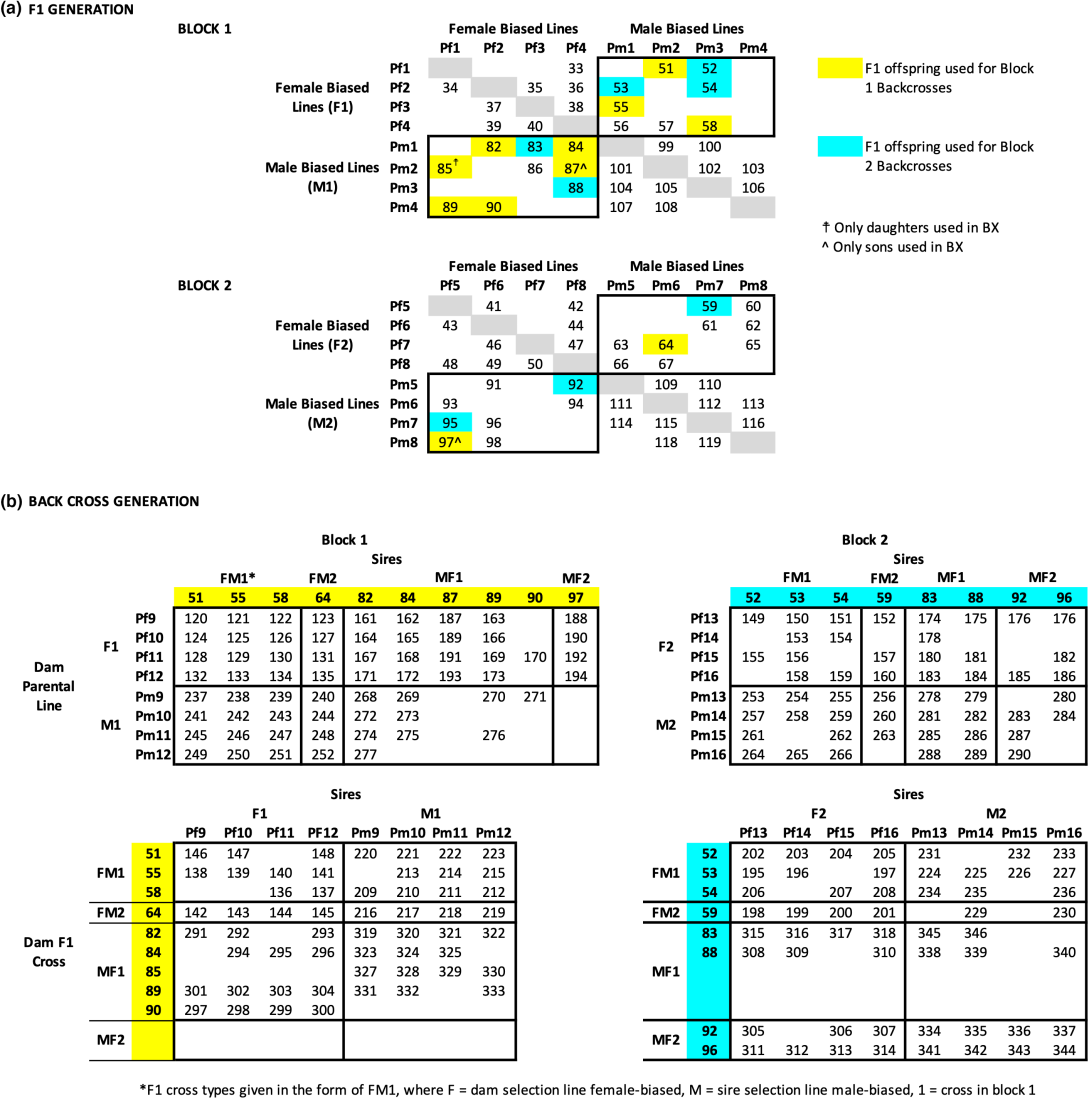
Schematic of crossing design. Parents came from sixteen broods (labeled Pm1‐8 and Pf1‐8) collected from male‐biased (M) or female‐biased (F) sex ratio lines created using truncation selection over six generations **a. F1 Generation**. Two blocks were used, each with four female‐biased lines (F1, F2) and four male‐biased selection lines (M1, M2). No within‐family crosses were done, and crosses were done only within blocks. Thus, for each FF and MM cross there were 12 possible crosses and for each FM and MF cross there were 16 possible crosses. Crossing cells filled with a number indicate we have data for a cross between these two lines. Only offspring from crosses between selection line types were carried forward for backcrosses; shaded cells indicate crosses whose offspring were used in backcrosses. **b. Backcross Generation**. Siblings from F1 crosses were backcrossed with individuals from the original male‐ and female‐biased lines. F1 cross numbers come from numbers identifying crosses in F1 Generation. Backcrosses used the same parental lines as for F1 crosses, at the next generation; backcross parental broods are labeled Pm9‐16 (from male‐biased selection line) and Pf9‐16 (from female‐biased selection line). Crossing cells filled with a number indicate we have data for that cross; empty cells are possible crosses within the subset of families used for which we did not collect data.

Line crosses were carried out between the two selection lines using offspring produced by the eight females (of >55 sampled) with the most biased brood sex ratio in each of the two selection lines (omitting any families with <6 individuals of either sex). Offspring from these 16 families were mated in an incomplete diallel cross design among four families (within family crosses were not done) from each line, replicated twice (Figure 1a).

### 2.1 F1 crosses

Crosses were done by taking individual males and females from the appropriate families and placing them together in the well of a 12‐well culture plate filled with 5 mL of filtered sea water (FSW). Males were removed after 7 days, and each female was subsequently checked twice daily for the presence of a mature (red) egg sac. Females with red egg sacs were placed on moist filter paper and the egg sac gently removed using a fine insect pin (Burton, 1985). The egg mass was placed into an empty well of a 6‐well culture plate filled with 10 mL of FSW. Females were returned to their home well and continued to be checked for an egg sac; for 50 of the 73 F1 crosses a second ripe egg sac was removed, and sex ratio estimates were calculated for the second brood as we hoped to estimate variance between broods within crosses in our analysis. Females typically have anywhere from 3–6 broods; all eggs are fertilized from sperm stored by the female during a single mating (Burton, 1985).

When the brood of egg sacs placed in 6‐well plates reached the copepodid stage (10–12 days post‐hatching), copepodids were isolated from one another by transferring individuals to an individual well in 24‐well plate filled with 2.5 mL FSW. At maturity, the number of males and females in each brood was counted to estimate BSR.

For F1 crosses, we divided lines into two blocks, each with four female‐ (F) and male‐biased (M) families, so that within each block there were 12 possible crosses of both FF and MM parents (within family crosses were not done, so 16 – 4 = 12 within line crosses) and 16 possible crosses for both MF and FM parents, for a total of 56 possible F1 crosses per block and 112 possible F1 crosses total. The actual number of crosses per cross type x block was 8–10; 71 F1 crosses that produced a minimum of 12 offspring for BSR estimate were analyzed (Figure 1a).

### 2.2 Backcrosses

Of the 71 F1 crosses analyzed, 34 were crosses between families with parents from two different selection lines (FM1, MF1, FM2, MF2; Figure 1). We thus had 34 F1 families from which we could set up backcrosses against eight families from the two selection lines, for a possible 272 × 2 = 544 backcrosses; we were able to set up backcrosses from 19 of our F1 families using four to eight male‐ and female‐biased sex ratio parent broods (using the next generation of the original selection lines, i.e., generation 8, and again using offspring of the eight females from each line with the most biased brood sex ratios) for a total of 223 backcrosses (Figure 1b). Offspring from all but three of these F1 families were used as both sires and dams, generating reciprocal backcrosses.

The first mature egg sac of each female in a backcross was plated into a 6‐well plate, as above. For some crosses, we also plated a second egg sac. Offspring of these crosses were allowed to mature in 6‐well plates and sex ratio determined when the brood matured by anesthetizing all individuals in the brood using 10% MgCl_2_ (in FSW) prior to counting males and females.

### 2.3 Statistical analysis

Phenotypic means and variances of brood sex ratio for each cross were calculated. We then tested whether the observed variance in sex ratio differed significantly (under‐ or over‐dispersion) from that of the expected binomial distribution by generating random numbers of males and females in each observed brood (keeping observed brood size), as recommended by Krackow et al. (2002) and modifying code from Postma et al. (2011).

A pedigree analysis (the ‘animal model’; Postma et al., 2011; Wilson et al., 2010) was used to estimate variance components of sex in *Tigriopus*. We created a pedigree that included all 19,571 individuals sexed and used the R package ‘MCMCglmm’ (Hadfield, 2010) to fit a generalized linear mixed model and generate Bayesian posterior distributions for estimating additive genetic variance, variance due to maternal effects, and heritability for sex. We treated sex as a threshold trait, modeling it as controlled by an underlying (unobservable) continuous trait, referred to as ‘liability’, and the observed phenotype of individuals as male or female depends on whether the liability trait value is above (male) or below (female) some threshold value (Bennewitz et al., 2007; Damgaard & Korsgaard, 2006; de Villemereuil, 2018; Roff, 1996). Statistically this is accomplished using a generalized linear model with a link function. Given this transformation from the continuous liability trait to a threshold trait, heritability can be estimated both on the liability trait scale and the observed trait scale (see de Villemereuil, 2018 for full details). The threshold model is equivalent to a generalized linear model with probit link; however, each individual can only have one measure (it is either male or female) and therefore we cannot estimate residual variance and must instead fix it at some arbitrary value in the model (Postma et al., 2011); we used 1, finding this gave us the most well‐behaved model (other values led to increased autocorrelation). We started with a model with no fixed effects and individual and dam as random effects, with the link function set as family = threshold. Six chains were run in parallel, each with a burn‐in of 100,000, thinning of 5000 and 1 million iterations, for a total sample size of 1080. We estimated effective sample size in all Bayesian models run to confirm validity of models. After ensuring chains were well‐mixed, data from all chains were combined and the R Package QGglmm (de Villemereuil, 2018; de Villemereuil et al., 2016) used to obtain heritability on the observed scale.

## 3 RESULTS

We originally hoped to include estimates of variance in brood sex ratio within parents and between broods. However, only 68 of 332 crosses (32 parent families, 73 F1, 227 backcross) had data for a second brood and we thus used only one brood from each cross in all analyses to avoid unequal sample size issues. Where two broods were available, we used the brood with the largest size (typically, but not necessarily, the first produced). To avoid imprecise estimates of brood sex ratio, we kept only crosses with brood size >11 for analyses; this led to eight crosses being dropped for a total sample size of 326 crosses (32 parent families, 71 F1 and 223 backcross; Figure 1).

**FIGURE 2:**
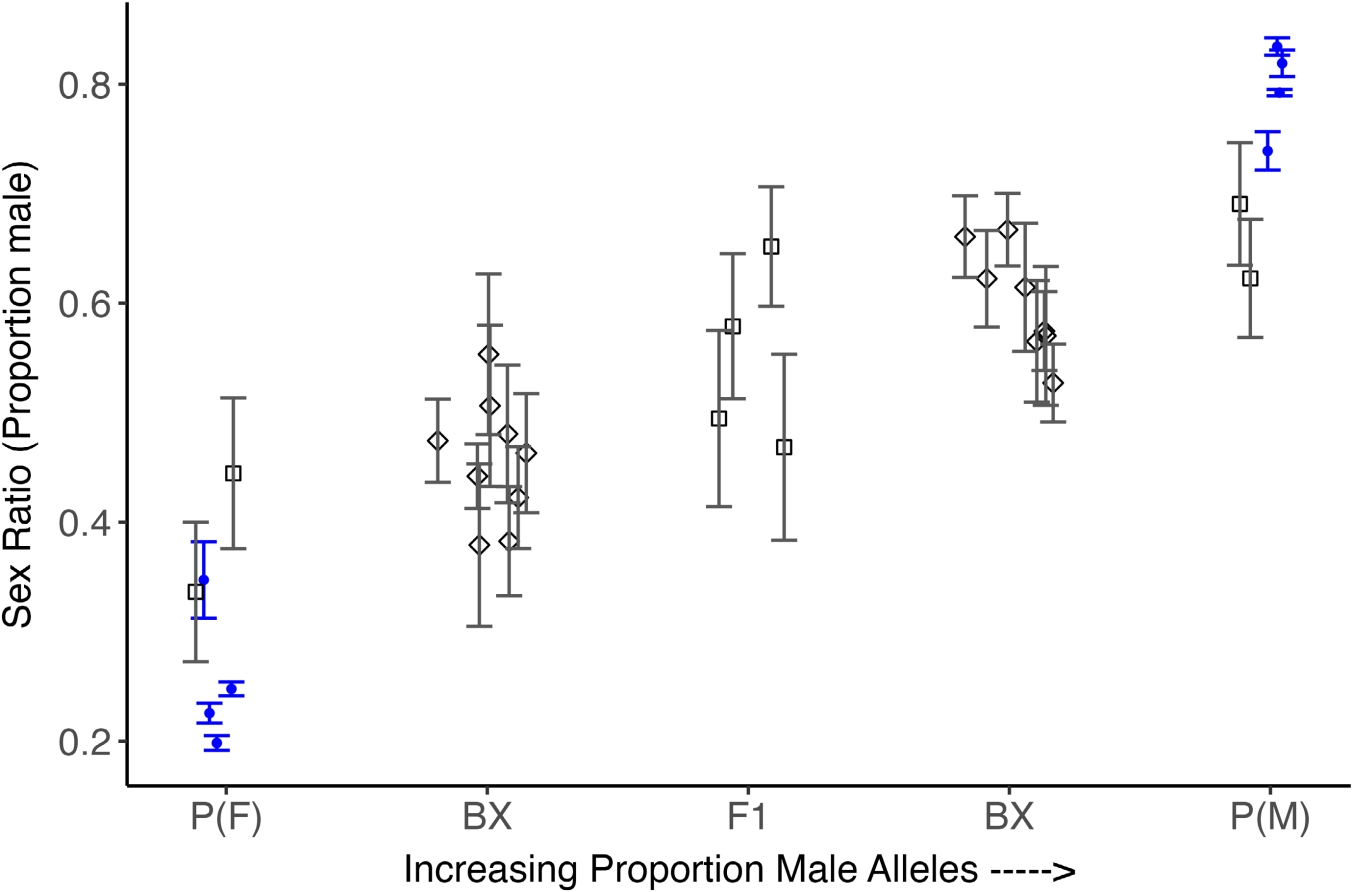
Mean and standard errors for brood sex ratios for all cross types done. Solid points are parent families from the selection (female‐biased selection on left‐hand side; male‐biased selection on right‐hand side); each point represents block by parent/backcross parent group. Note that parents and backcross parents (shown in blue) were based on selected individuals; the eight most biased sex ratio families in each selection line. Only results from crosses where *n* > 5 for each cross type by block combination are plotted. White squares = F1 crosses, white diamonds = backcrosses.

Phenotypic variance increased substantially within one generation, even for parents within the same selection type and block. In the F1 generation, phenotypic variance in F1 offspring was similar regardless of whether parents both came from the same selection type (i.e., both male‐biased or both female‐biased) or different section types (i.e., dam was from male‐biased line and sire was from female‐biased line or vice versa); this was true for both blocks (Table 1). In addition, phenotypic mean values for F1s and backcrosses fell at the midpoints of parent families and of parent and F1 families, respectively, as expected for a polygenic inherited trait (Figure 2).

**TABLE 1:** Phenotypic variance in brood sex ratio increases across one generation of reciprocal crossing of dams and sires from selected lines whether lines were from the same selection type (FF, MM crosses) or different selection types (FM, MF crosses).

		Parent generation	F1 generation	F1 cross type
Block 1	Female‐biased lines	0.000435 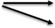	0.0325	FF
			0.0520	FM
	Male‐biased lines	0.003228 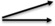	0.0267	MF
			0.0312	MM
Block 2	Female‐biased lines	0.004981 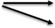	0.04267	FF
			0.06566	FM
	Male‐biased lines	0.000470 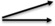	0.03125	MF
			0.02909	MM

**FIGURE 3:**
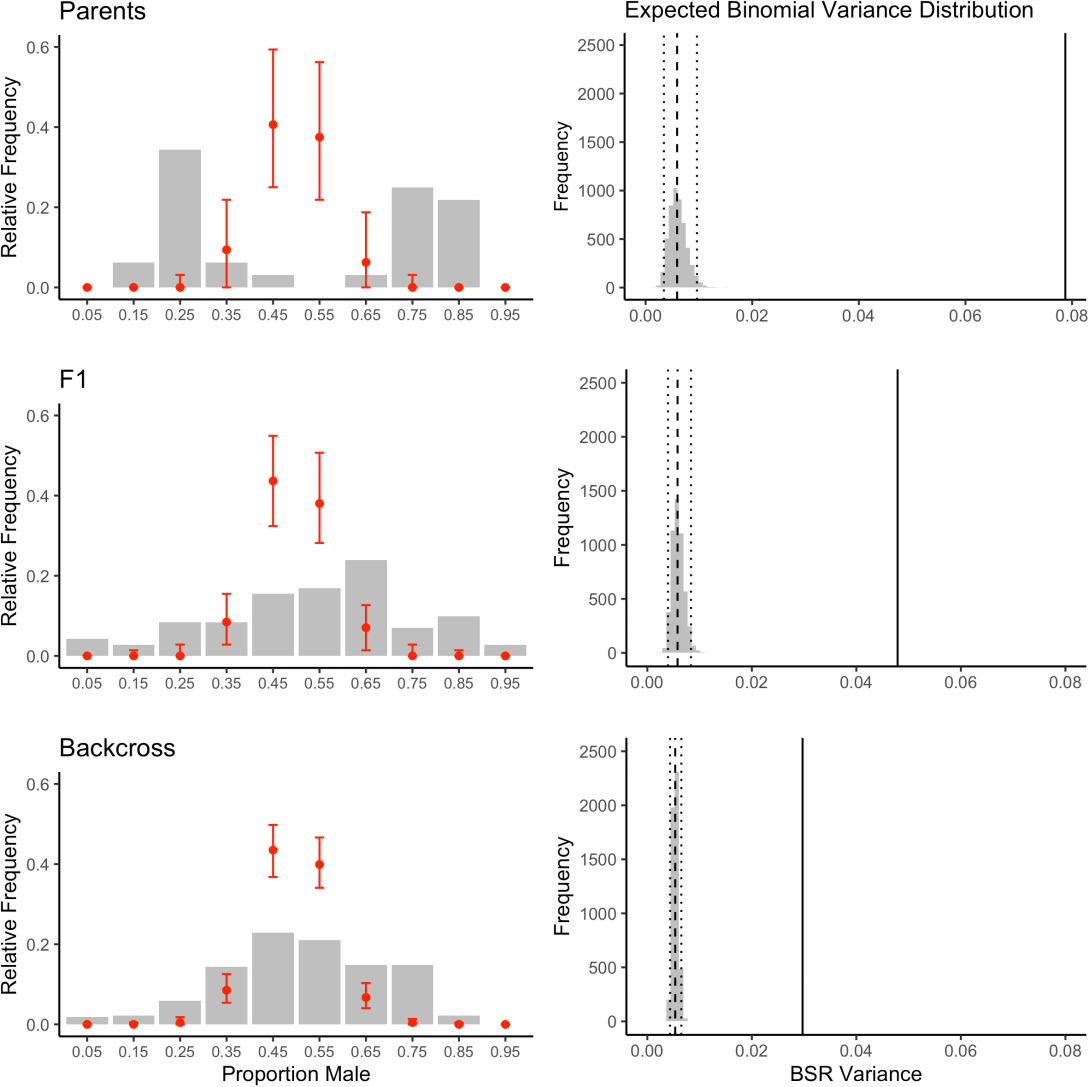
Observed (bars) and expected (points ± SE) distribution of brood sex ratio (left panel) and expected brood sex ratio (BSR) distribution (right panel) for parental, F1 and backcross generations of *Tigriopus californicus* crosses among male‐ and female‐bias selection lines. On right panel median and 95% quantile limits are shown for expected distribution of variance using dashed and dotted vertical lines, respectively; solid lines indicate observed BSR variance.

The observed variance in brood sex ratio using all crosses combined was 0.038 and significantly outside the bounds of binomial expected variance (randomization median variance = 0.0055; 95% CI: 0.0046–0.0065); in 5000 simulations a variance as large as that observed did not occur (Figure 3). This result clearly indicates that sex is not inherited as a simple dichotomous trait, as is the case in organisms with a sex chromosome.

In addition, brood sex ratio distribution by generation data show that while variation decreases in brood sex ratio as families are crossed and backcrossed, the observed variance in brood sex ratio far exceeds the range of the expected variance based on a null model of binomial trait inheritance and controlling for observed brood sizes (Figure 3). Similarly, while the brood sex ratio distribution goes from bimodal to unimodal with increasing generations, the curve remains flattened with fewer observations of brood sex ratio having 0.4 to 0.6 proportion male than expected and more with proportion male <0.4 and >0.6 than expected under binomial inheritance (Figure 3).

### 3.1 Pedigree analysis

We first fit a pedigree‐based model with the random effects of animal and dam using MCMCglmm. This random effects‐only model allowed us to confirm the model fit appropriately and returned the correct overall mean sex ratio. We used a threshold model, as is standard for binary or count trait values, modeling heritability on the underlying liability trait (de Villemereuil, 2018) and achieved a model with good mixing of chains and acceptable autocorrelations (the majority <0.10; one of the six replicate chains had high autocorrelations of 0.18 and 0.19; removal of this chain from analysis did not affect analysis outcome). From this model, mean heritability on the liability scale was 0.237 (95% credible interval (CI): 0.00–0.51). Using QGglmm (de Villemereuil et al., 2016) we estimated trait values on the observed scale and found heritability for sex on the observed scale was 0.150 (95% CI: 0.0–0.33).

We next fit a model that included fixed effects of block and parental selection origin (i.e., are maternally and paternally inherited genes from male‐biased (M) or female‐biased (F) selection lines; four levels were possible: FF, FM, MF, MM, where the first letter indicates maternal gene source, and second letter indicates paternal gene source). No effect of block was present, but paternal by maternal combinations of male‐biased or female‐biased population origin did differ, with pMCMC <0.006 for each group relative to the reference group of female‐biased lines in both paternal and maternal ancestry. We calculated heritability on both scales for this model integrating the variance from fixed effects to ensure our estimate is not inflated by the concomitant decrease in residual variance when fixed effects are partitioned out (de Villemereuil, 2021; de Villemereuil et al., 2018). Heritability on the liability scale was 0.272 (95% CI: 0.136–0.437). On the observed scale, heritability for sex was 0.09 (95% CI: 0.034–0.143). Thus, accounting for the variance due to selection line differences did not change the heritability much but did substantially tighten up our credibility interval and provide us with a better estimate of heritability.

## 4 DISCUSSION

In this study we have used strongly selected, highly inbred biased sex ratio lines to assess heritability of sex in a marine copepod species, *T. californicus*, without sex chromosomes using an incomplete diallel cross. We show: (1) Substantive extra‐binomial variation for sex that persists through two generations of random crosses in a controlled laboratory environment; (2) Mean phenotypic values for sex ratio in F1 and backcrossed offspring match the midpoint of the parental values as predicted in a normally distributed polygenic trait with many genes of small effect; (3) Heritability for sex (on the observed scale) of 0.09 and heritability for the underlying threshold trait of 0.271. Heritability estimates for binary traits are necessarily limited to lower values due to the nonadditive effects created by the link function and it is unclear what the maximum observable heritability is for sex in *T. californicus*.

**FIGURE A1:**
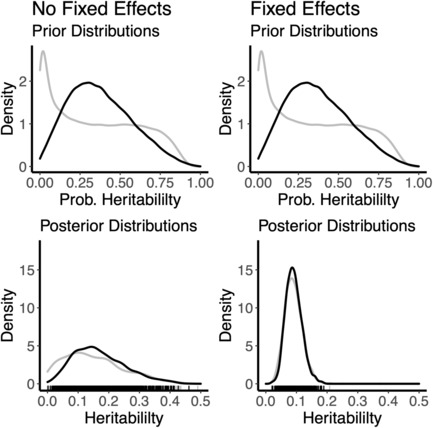
Result of changing prior distribution used from uninformative (gray line) to informative (black line) (top panel) on posterior distribution of heritability on the observed scale in sex of an individual (bottom panel). The informed prior slightly decreases the width of the posterior distribution (shown in black; gray line is density for uninformative prior). UP = uninformative prior; IP = informative prior.

*Tigriopus californicus* gives every indication of having true polygenic inheritance of sex, with many genes of small effect contributing to the underlying liability trait value for the threshold trait of sex. The data presented in the current study confirms previous work done on sex ratio in this organism using different populations and estimation methods (Alexander et al., 2014, 2015; Foley et al., 2013; Voordouw et al., 2005, 2008; Voordouw & Anholt, 2002b). Using a Bayesian pedigree analysis (i.e., the animal model), we show that the tendency for an individual *T. californicus* to be male has a significant genetic component, with a heritability estimate of 0.09, after removing variance due to fixed effects of breeding lines and blocks. Note that in this study, breeding lines were different isofemale lines and thus clearly also included a genetic component; thus, our heritability estimates are likely to be underestimates. By including the fixed effect of parental selection lines in our Bayesian model, the credible interval of our estimate of heritability was decreased to one‐third the size (Figure A1). This matches previously published estimates that were 0.31 for mother–offspring heritability estimates (Voordouw & Anholt, 2002b) and realized heritability estimates (Alexander et al., 2014), but ranged from 0–0.24 for full‐sibling estimates (Voordouw & Anholt, 2002b). We initially ran the model using a standard uninformative prior distribution, but as we had estimates of heritability from previous studies (Alexander et al., 2014; Voordouw & Anholt, 2002b), we were in a position to use an informed prior and Bayesian analysis to update our prior knowledge. Using an informed prior did allow us to minimally reduce credible intervals around our heritability estimates (by 4.8% in model with fixed effects) but did not affect analysis results and had far less of an effect than adding in ancestral selection line information (Figure A1).

**FIGURE 4:**
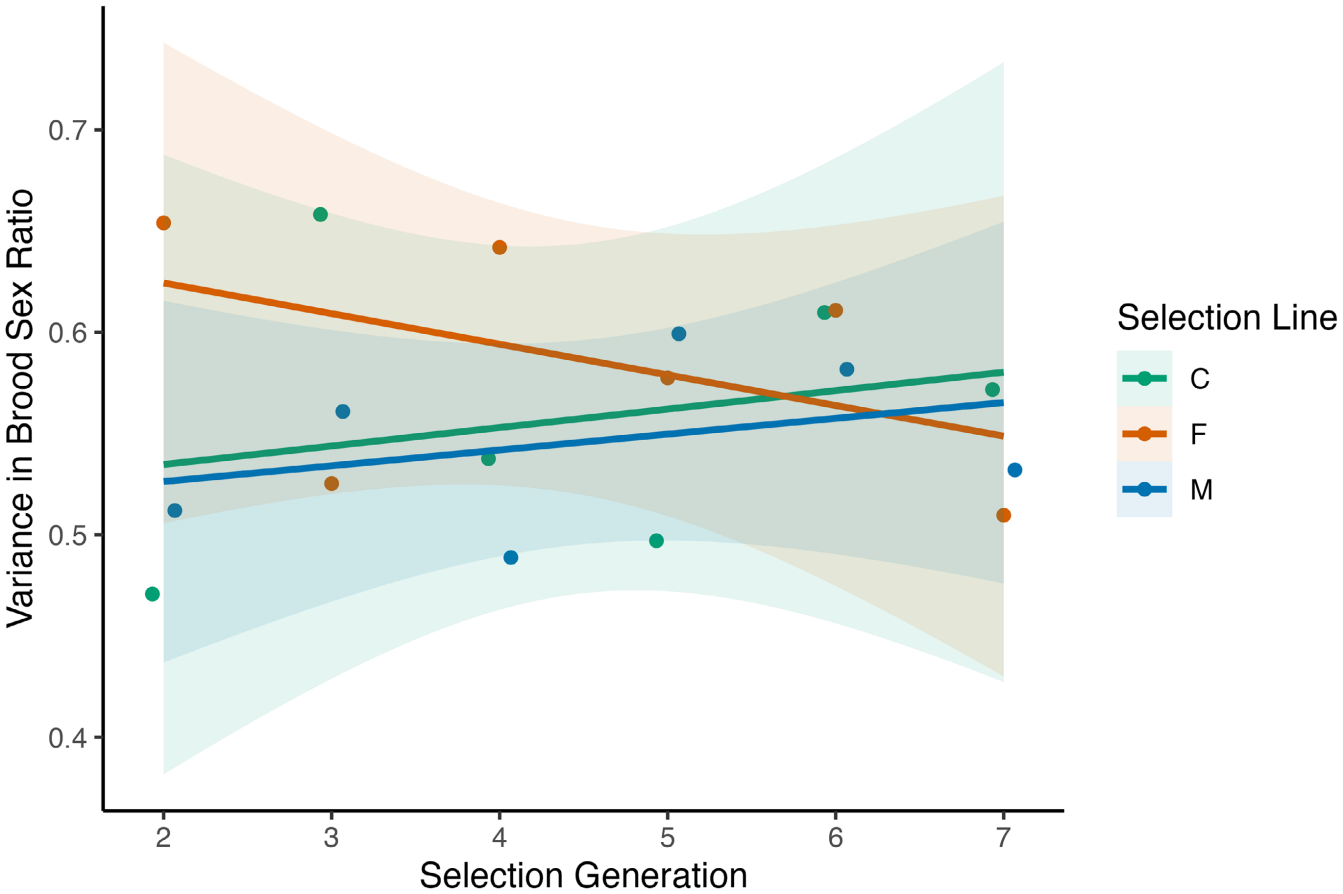
Change in phenotypic variance for brood sex ratio, measured as standard deviation in qnorm(proportion male) for each generation during truncation selection for biased brood sex ratios. Data from (Alexander et al., 2014). Selection lines are: C = control (no selection), F = female‐biased, M = male‐biased. Under strong artificial selection phenotypic variance is expected to decrease; standard least‐squares lines of best fit are given for each line with shading indicating 95% regions to highlight that such decrease in variance did not occur. After generation 7, brood sex ratio (proportion male) was 0.75 for M line, 0.35 for F line, and 0.45 for C line; all had brood sex ratio = 0.51 at generation 1.

The maintenance of variability, and continued presence of extra‐binomial variation, when crossing offspring of two individuals from the same selection line provides strong evidence for many genes of small effect controlling sex determination in *T. californicus*. Further, the similar increase in phenotypic variation for F1s that occurred when different families from the same or different selection type populations were crossed suggests selected families within the same selection line were achieved using different genes. If families were genetically similar, we would not expect much change between crosses within each selection type by block (Table 1). We think this is also why F1 crosses within selection types have reduced sex ratio bias relative to the parents—crosses between families disrupted gene complexes formed within families during selection (Figure 2). This result is further corroborated through consideration of the phenotypic variance in brood sex ratio across generations under selection for biased sex ratios. While the sex ratios responded strongly to selection, the variance in brood sex ratio was essentially unchanging over the seven generations of selection (Figure 4). This also matches observations in the field of extensive variance in brood sex ratio both within and among sites (Voordouw et al., 2008) and models showing polygenic sex determination is maintained indefinitely when combined with seasonal fluctuations of alternating selection (Bateman & Anholt, 2017).

Several other aspects of *T. californicus* biology are likely to contribute to maintenance of genetic variance in the species. *Tigriopus* live in supralittoral marine splash pools that are both ephemeral and highly variable environments and form a complex metapopulation, with each splash pool representing a subpopulation and migration occurring between splash pools (Dybdahl, 1994). Charnov and Bull (1989) demonstrated that in patchy environments, if females do relatively better in one patch, then the primary sex ratio is male‐biased (the sex coming from the poorer habitat). In addition, environmental sex determination (ESD) is also known to play a role in *Tigriopus* sex determination (Voordouw & Anholt, 2002a). In contrast, the failure of *T. californicus* to develop heterogametic sex determination is perhaps surprising given that females have achiasmatic meiosis (Ar‐Rushdi, 1963), and with achiasmata in one sex any sex‐determining gene that evolves should quickly lead to differentiated sex chromosomes (Wright et al., 2016).

A recent simulation by Butka and Freedberg (2019) reveals that when environmental sex determination is present and controlled by many loci (≥10), limited dispersal rates (<0.5) among multiple subpopulations lead to a male‐biased sex ratio equilibrium due to local adaptation to different developmental environments. Population genetic studies do suggest that *Tigriopus* dispersal among rocky outcrops is limited (Burton & Feldman, 1981). At least six QTL exist for sex determination (Alexander et al., 2015), and recent research suggests such QTLs likely represent many separate genes each (Walsh & Lynch, 2018). In nature, *T. californicus* populations do tend to be male‐biased (Voordouw et al., 2008). The combination of the modeling and field data thus suggest one possible explanation for male‐biased sex ratios observed in *T. californicus* and further reinforces the idea that the species has polygenic sex determination. Environmental variance represents only a minor portion of sex ratio variance in *T. californicus* (Voordouw & Anholt, 2002a, 2002b) and genetic influence on pivotal temperature has not been considered; it is possible that selection for sex ratio bias is in fact selecting for changes in pivotal temperature (Wright et al., 2016).

Variance for threshold traits on the observed scale contains additional variance and this reduces maximum heritability. For example, while the liability trait, on the latent scale, has a continuous range of values, the observed phenotype has only one of two values, determined by the latent scale breeding value and the threshold value. Thus, if the threshold value is 0.4, whether the individual's breeding value is 0.2 or 0.01 they will be male on the observed scale. This has the effect of increasing the nonadditive genetic variance for the trait on the observed scale, thereby limiting the maximum heritability possible. In particular, heritability on the observed scale will always be lower than that on the latent scale (de Villemereuil, 2020; de Villemereuil et al., 2016; Dempster & Lerner, 1950). This is one reason why the heritability observed here, given on the observed scale, is lower than the realized heritability estimated on the latent scale by Alexander et al. (2014). Nonetheless, a strong response to truncation selection for biased sex ratios clearly indicates some aspect of sex determination in the species is sufficiently heritable to respond to selection. We speculate that epistatic effects may also limit our ability to estimate true heritability.

The difference in estimated heritabilities may also reflect violation of any one of the many assumptions of the threshold model. In particular, the model assumes allelic effects at the many loci contributing to liability are multivariate normal. This is both unlikely to be true and difficult to assess. Benchek and Morris (2013), using simulated data to test heritability estimates when true liability included a common environmental effect that was not normally distributed, found that heritability estimates can be highly biased in this case and that the direction of bias was not consistent. The model also assumes no pleiotropic or epigenetic effects, but environment is known to influence sex determination in *Tigriopus*. Temperature effects on sex may well be influenced by genes and alleles affecting sex determination and temperature effects on sex determination seem likely to interact with each other as well as with the environment. At the heart of the challenge is that selection acts on the multivariate phenotype and any one component in isolation may have low heritability although the combined traits have high heritability (Walsh & Lynch, 2018).

Regardless of the underlying genetic mechanism, it seems likely that the complex metapopulation dynamics of *Tigriopus* (Burton & Swisher, 1984; Dethier, 1980; Johnson, 2001; Powlik, 1999) may be an important component to understanding the unusual maintenance of polygenic sex determination in the species. The highly unpredictable nature of the splash pools these copepods inhabit may further provide insight into why this species has failed to evolve a single gene of large effect for sex tendency. Pools that are washed out by wave action will cause large‐scale mortality unrelated to phenotype, as most individuals washed into the ocean are likely to be consumed by fish (Dethier, 1980).

While the presence of multiple genes affecting sex has recently been observed in many animals, particularly in fish species (Martínez et al., 2014), in most of these cases, a sex chromosome or gene with large effect on sex is present in the species. The case for polygenic sex determination has perhaps been most strongly made for the model organism zebrafish (Liew et al., 2012), where only two to three (depending on strain) sex‐determining regions (compared to six in *Tigriopus*) have been identified in domesticated zebrafish (Wilson et al., 2014), and wild zebrafish have a ZZ/ZW sex determining system (Wilson et al., 2014). Similarly in European sea bass, while genetic components for sex determination are present and suggest polygenic sex determination (Vandeputte et al., 2007), sex determination is also strongly influenced by temperature and wild populations do not show the same sex ratio biases seen in farmed populations (Vandeputte et al., 2012). We suggest that *T. californicus* represents a unique polygenic system in that there is no indication that any one gene has a large effect on sex determination nor that such a gene has ever existed in the species. The species thus continues to present an interesting case study that appears to defy theoretical expectations.

## AUTHOR CONTRIBUTIONS

**Jean Richardson:** Conceptualization (equal); formal analysis (lead); investigation (equal); methodology (equal); visualization (lead); writing – original draft (lead); writing – review and editing (equal). **Heather Alexander:** Conceptualization (equal); investigation (equal); methodology (equal); writing – review and editing (equal). **Bradley R Anholt:** Conceptualization (equal); funding acquisition (lead); methodology (equal); writing – review and editing (equal).

## CONFLICT OF INTEREST STATEMENT

All authors declare they have no competing interests.

## Data Availability

Raw data, R code for cleaning and formatting data, cleaned data and R code for analyses are available on Dryad at https://doi.org/10.5061/dryad.mpg4f4r4k.
